# Ex vivo 100 μm isotropic diffusion MRI-based tractography of connectivity changes in the end-stage R6/2 mouse model of Huntington’s disease

**DOI:** 10.1002/nep3.14

**Published:** 2022-12-20

**Authors:** Ashwinee Manivannan, Lesley M. Foley, T. Kevin Hitchens, Ivan Rattray, Gillian P. Bates, Michel Modo

**Affiliations:** 1 Department of Neuroscience, University of Pittsburgh, Pittsburgh, Pennsylvania, USA; 2 Animal Imaging Center, University of Pittsburgh, Pittsburgh, Pennsylvania, USA; 3 Department of Neurobiology, University of Pittsburgh, Pittsburgh, Pennsylvania, USA; 4 Department of Neurodegenerative Disease, Queen Square Institute of Neurology, Huntington’s Disease Centre and UK Dementia Research Institute at UCL, University College London, London, UK; 5 Department of Radiology, University of Pittsburgh, Pittsburgh, Pennsylvania, USA; 6 Department of Bioengineering, University of Pittsburgh, Pittsburgh, Pennsylvania, USA; 7 Center for the Neural Basis of Cognition, University of Pittsburgh, Pittsburgh, Pennsylvania, USA

**Keywords:** connectome, diffusion tensor imaging, Huntington’s disease, mouse, MRI, tractography

## Abstract

**Background:**

Huntington’s disease is a progressive neurodegenerative disorder. Brain atrophy, as measured by volumetric magnetic resonance imaging (MRI), is a downstream consequence of neurodegeneration, but microstructural changes within brain tissue are expected to precede this volumetric decline. The tissue microstructure can be assayed non-invasively using diffusion MRI, which also allows a tractographic analysis of brain connectivity.

**Methods:**

We here used ex vivo diffusion MRI (11.7 T) to measure microstructural changes in different brain regions of end-stage (14 weeks of age) wild type and R6/2 mice (male and female) modeling Huntington’s disease. To probe the microstructure of different brain regions, reduce partial volume effects and measure connectivity between different regions, a 100 μm isotropic voxel resolution was acquired.

**Results:**

Although fractional anisotropy did not reveal any difference between wild-type controls and R6/2 mice, mean, axial, and radial diffusivity were increased in female R6/2 mice and decreased in male R6/2 mice. Whole brain streamlines were only reduced in male R6/2 mice, but streamline density was increased. Region-to-region tractography indicated reductions in connectivity between the cortex, hippocampus, and thalamus with the striatum, as well as within the basal ganglia (striatum—globus pallidus—subthalamic nucleus—substantia nigra—thalamus).

**Conclusions:**

Biological sex and left/right hemisphere affected tractographic results, potentially reflecting different stages of disease progression. This proof-of-principle study indicates that diffusion MRI and tractography potentially provide novel biomarkers that connect volumetric changes across different brain regions. In a translation setting, these measurements constitute a novel tool to assess the therapeutic impact of interventions such as neuroprotective agents in transgenic models, as well as patients with Huntington’s disease.

## 1 | INTRODUCTION

Motor, cognitive, and neuropsychiatric symptoms develop in Huntington’s disease (HD) due to neuronal loss that is caused by a cytosine, adenine, guanine (CAG) repeat expansion in the huntingtin (*HTT*) gene.^1^ Although a genetic test provides certainty of the disease, the development of symptoms is difficult to predict and associated with changes in brain anatomy due to a progressive neurodegeneration. Visualizing and measuring these changes is hence important to chart the progression of the disease state, but also potentially provides diagnostic value to identify a prodromal stage that precedes symptom development and will be key to assess neuroprotective strategies.^2^ Considering the variance in symptoms between patients, measuring changes in brain structure and function can objectively define disease burden and how this is modified longitudinally using treatment interventions.^3,4^ Ideally, noninvasive imaging provides quantifiable in vivo biomarkers that can be used for disease management.^5,6^

Magnetic resonance imaging (MRI) has emerged as a versatile and objective tool to evaluate noninvasively neuropathological brain changes in patients with HD.^5^ Atrophy in the cortex and the caudate-putamen emerge as early biomarkers of the disease condition, with some changes in structure preceding symptom onset. A high correlation between symptoms and MRI biomarkers has been found to be consistent with disease progression.^7^ Diffusion MRI is sensitive to subtle changes in the tissue microenvironment. There is further evidence of microstructural changes, measurable using diffusion MRI, that anticipate volumetric changes.^8,9^ For instance, mean diffusivity (MD) increases with neuronal loss. Neuronal loss also invokes a decrease in connectivity that is likely to drive downstream volumetric changes.

Connectivity and network topology analyses reveal disturbances that precede symptom onset in HD.^10–13^ Longitudinal monitoring of connectivity could hence be used as a predictive tool of disease progression.^11^ Structural connectivity changes precede alterations in functional connectivity,^14^ especially along the cortico-basal ganglia network.^15^ Although there is some evidence that frontal cortex-striatal circuits are affected long before the disease manifestation,^16,17^ there is a high degree of temporal and spatial variability in cortical neurodegeneration.^18^ Changes in regional connectivity describe a network of symptom-independent changes associated with different stages of the disease and are hence increasingly being explored as potential biomarkers that could report on disease-modifying treatments.

As therapeutics are typically evaluated first in animal models of HD, it is essential to ensure that the same biomarkers, such as MRI, can be used in a translational framework.^19^ A variety of animal models have been developed to mimic the neuropathology of HD, but transgenic mouse models exhibiting a progressive neurodegeneration carry the highest face validity.^20^ Although extensive characterizations of transgenic mouse models of HD have demonstrated construct validity for behavioral deficits and in vivo atrophy of brain structures as measured by MRI, as well as histopathology,^21–26^ very few studies have so far investigated diffusion changes in microstructure or tractography-based connectivity changes.^27–38^ Although in vivo longitudinal measurements are ideal to evaluate the progression and impact of treatments on neurodegeneration-induced connectivity changes, diffusion tensor imaging (DTI) requires extended image acquisition times that limit the isotropic spatial resolution required for tractography of connections in the mouse brain.

To achieve a high-quality isotropic resolution suitable for tractography of the individual structures within the basal ganglia using diffusion MRI, protracted scanning times are required, which are amenable to ex vivo investigations in end-stage (14 weeks old) R6/2 transgenic mice modeling HD. To measure brain atrophy, *T*_2_-weighted volumetric MR images were acquired, as well as DTI, which quantified scalar indices to measure changes in tissue microenvironments and afforded a tractographical analysis of connectivity between regions of interest (ROI). This proof-of-principle study demonstrates the utility of DTI to analyze the connectivity of different brain regions in neurodegenerative conditions and to provide a translational measure to characterize disease progression and evaluate novel treatment strategies.

## 2 | METHODS

### 2.1 | Animals

All procedures were performed in accordance with the *Animals* (Scientific Procedures) *Act 1986* and were approved by the King’s College London Ethical Review Process Committee. Wild-type (WT) (*n* = 8: *n* =4 female; *n* = 4 male) and R6/2 transgenic mice (*n* = 8: *n* = 4 female; *n* = 4 male) were bred, genotyped, and CAG repeat sized at 14 weeks of age, as previously described.^24^ Repeat length variance for R6/2 mice was kept at a minimum and did not differ for male (range 203–209, mean 206) and female (range 203–210, mean 207) mice. Perfusion-fixation occurred under terminal anesthesia (Euthatal; Marial Animal Health) with heparinized saline flushing out blood before tissue fixation with 4% paraformaldehyde (Pioneer Research Chemicals). After perfusion, heads were severed and postfixed overnight before being immersed in phosphate-buffered saline (PBS) for MRI.

### 2.2 | MRI

For MR imaging, mouse heads were immersed into proton-free FluorInert (Sigma) in a syringe to immobilize the head for long scanning times at a high resolution, where minimal movement between gradient directions can affect image quality. Images were acquired with a Bruker AV3 HD 11.7T/89 mm vertical bore microimaging system, with a Micro 2.5 gradient insert (capable of up to 150 G/cm) in a 25 mm quadrature birdcage coil (Bruker). Sample temperature was maintained at 21 ± 0.1°C with a Bruker SmartCooler BCU-1 40/50 air chiller and probe heater with a thermocouple feedback loop.^39^ Scanning parameters were defined in ParaVision 6.0. A 3D spin-echo diffusion MRI scan was acquired (repetition time, TR = 700 ms, echo time, TE = 24 ms, diffusion time = 12 ms, diffusion encoding duration 5 ms, *b* value = 1401.57 s/mm^2^, with 12 non-collinear diffusion directions and one *A*_0_ image, number of averages, NA = 1, matrix 196 × 128 × 128, 100 μm isotropic resolution, and 31 h 3 min acquisition time), followed by a coregistered 3D *T*_2_-weighted spin-echo scan at the same resolution (TR = 3500, TE = 40, NA = 1, and 15 h 55 min acquisition time). We used a 3D spin-echo diffusion sequence to achieve high isotropic spatial resolution for investigating connectivity between small anatomical structures in the mouse. This approach is time-consuming, which limits the diffusion paradigm, but we chose this method over an EPI approach to avoid potential image distortion from susceptibility artifacts at high field.

### 2.3 | MR image processing

Diffusion MR images were processed using Diffusion Spectrum Imaging (DSI) studio (available at: http://dsi-studio.labsolver.org/dsi-studio-download).^40^ The brain was masked, and the rest of the head was removed as background before processing. Reconstruction of the DTI was achieved by performing an Eigenvector analysis on the calculated tensor.^41^ Mean (MD), radial (RD), and axial diffusion (AD) maps were computed for measurement of scalar indices, as was fractional anisotropy (FA). Streamlines reflecting connectivity were mapped for the whole brain using a multidirectional deterministic fiber tracking algorithm using 10 seeds/voxel with random subvoxel positioning of seeds.^39,40^ Trilinear interpolation in all orientations with a step size of 0.05 mm (1/2 voxel length) was performed with an FA of 0.02 serving as a tracing endpoint of streamlines at the end of the sample, as fiber tracing was not restricted to only white matter. A streamline length of 0.2 mm (2× voxel length) was considered the minimum length of streamlines, with a maximum length of 10 mm (15 mm sample sagittal length) and an angular threshold of 60° being used to end connectivity tracing. Total number of streamlines for whole brain and individual ROIs were recorded to calculate a streamline density (streamlines/mm^3^) for group comparisons. Streamline length was also recorded, as there is some evidence that it is associated with regional atrophy in premanifest HD patients.^42^

### 2.4 | ROIs

**FIGURE 1:**
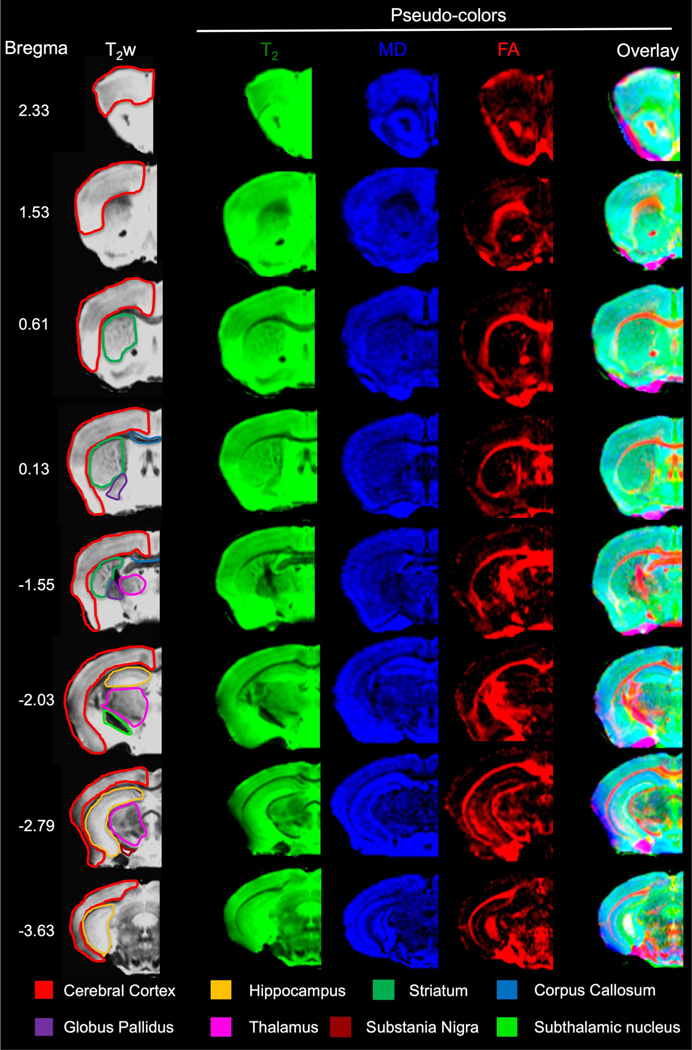
Anatomical segmentation of ROIs in Huntington’s disease. To investigate the differential effects of Huntington’s disease on CC, cortical areas, and HC, as well as structures of the basal ganglia, ROIs were manually defined in both control and R6/2 mice. A manual segmentation ensures that a differential atrophy of structures did not affect the anatomical definition of these ROIs. CC, corpus callosum; HC, hippocampus; ROIs, regions-of-interest.

ROIs were defined manually based on anatomical structures known to be affected by HD (Figure 1), notably whole cerebral cortex (Ctx), motor cortex (MC), somatosensory cortex (SMC), corpus callosum (CC), striatum (Str), globus pallidus (GP), nucleus accumbens (NAc), thalamus (Th), substantia nigra (SN), subthalamic nucleus (STN), hippocampus (HC), and olfactory bulb (OB). Anatomical structures were defined based on those defined in the standard mouse atlas.^43^

To establish if there was a differential loss of connectivity within the basal ganglia, point-to-point connections between ROIs (e.g., cortico-striatal tract) were also compared with “seeds” in one ROI (e.g., cortex) and those “ending” in another (e.g., striatum). To account for distance between ROIs, maximum streamline length was set to 10 mm for connections between cortex with the HC and striatum, 7 mm for streamlines between the thalamus and striatum, and 3 mm to map tracts from the striatum to the GP, GP to STN, and STN to SN, as well as SN to thalamus. We here analyzed both the left and right hemisphere, as there is evidence of brain asymmetry in manifest cases of HD, although this was not the case in premanifest HD.^44^

### 2.5 | Statistical analysis

Graphs were constructed using Prism version 9.1.2 (GraphPad Software). Two-way analyses of variance (genotype and gender as between-subject factors) were used to compare groups, with Šidák post hoc analyses establishing significant difference between individual groups. The significance level for these comparisons was set at *p* < 0.05 and is reported in the Results section. Detailed statistical results are summarized in Supplementary Tables 1–5. Using G*Power 3.1, an a priori power analysis (assuming a Cohen’s *f* effect size of 1.1) for brain volume, based on previous observations,^24^ required a total sample size of 14 subjects to achieve a 95% statistical power for comparisons of means across four experimental groups.

## 3 | RESULTS

### 3.1 | The volumes of cortical and subcortical structures are reduced in HD

End-stage HD affects many different anatomical regions and is reflected in a reduced volume of these structures. The entire Ctx here was significantly reduced in 14-week-old end-stage female R6/2 mice (*p* < 0.05); it was also reduced in male mice, albeit not significantly (Table 1, full statistical results can be found in Supporting Information: Table S1). This was also the case for the MC, but the SMC was reduced (*p* < 0.05) in both female and male R6/2 mice. A significantly (*p* < 0.05) reduced volume in both female and male R6/2 mice was observed in the striatum and STN. The volumes of the SN, GP, NAc, thalamus, and HC were only significantly reduced (*p* < 0.05) in female R6/2, whereas in male R6/2 the OB presented with smaller volumes (*p* < 0.05). The volume of the CC was equivalent between R6/2 and WT mice. End-stage HD, therefore, exerted widespread structural changes in the brains of both male and female subjects, with some changes being accentuated or attenuated by the subjects’ biological sex.

### 3.2 | Biological sex affects diffusion measurements in R6/2 Huntington diseased mice

In contrast to volumetric measurement, diffusion measurements reflect microenvironmental changes within the tissue irrespective of its volume. MD was reduced (*p* < 0.05) for male R6/2 in the Ctx, MC, SMC, striatum, SN, thalamus, HC, and CC (Table 2, full statistical results can be found in Supporting Information: Table S2). In contrast, in female R6/2, MD was increased (*p* < 0.05) in the striatum, NAc, STN, and OB. MD hence showed a major effect of biological sex. This pattern of increases for females and decreases for males was also observed for RD and AD (Table 2), but no significant effects on FA were observed (Table 3, full statistical results can be found in Supporting Information: Table S3).

### 3.3 | Brain connectivity

**FIGURE 2:**
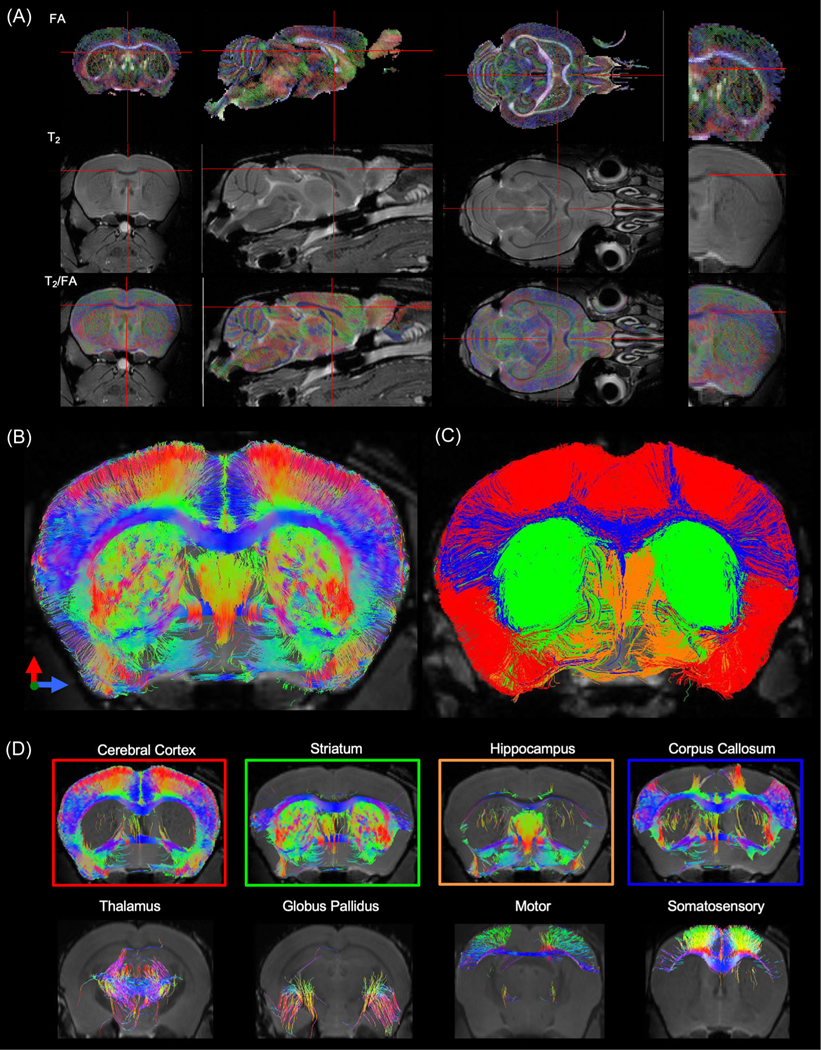
Diffusion directionality and tractography. (A) DEC images overlaid on *T*_2_-weighted structural images reveal the directionality of individual voxel water diffusion in the R6/2 mouse brain. (B) Exploiting this diffusion directionality, streamlines tracking fibers connecting different parts of the brain can inform on brain connectivity. (C) Parcelation of connectivity based on the origin of streamlines probes regional connectivity with fibers originating in the Ctx (red), striatum (green), HC (orange), or CC (blue). (D) Separation of the regions with streamlines colored for fiber direction provides a deeper understanding of how these regions and their connectivity are impacted by Huntington’s disease. CC, corpus callosum; DEC, diffusion-encoded color; HC, hippocampus.

DTI measures the directionality of water diffusion within a single voxel and this can be represented by a diffusion-encoded color (DEC) map (Figure 2A), using the principle axis of diffusion as the color hue with brightness representing increasing FA. FA allows the tracing of water movement along axonal tracts to compute streamlines that visualize brain connectivity (Figure 2B). Tracing connections based on their region of origin affords a region-by-region analysis of brain connectivity (Figure 2C). Seeding of streamlines in different brain regions afforded a segmentation of brain connectivity based on the origin of these (Figure 2D).

**FIGURE 3:**
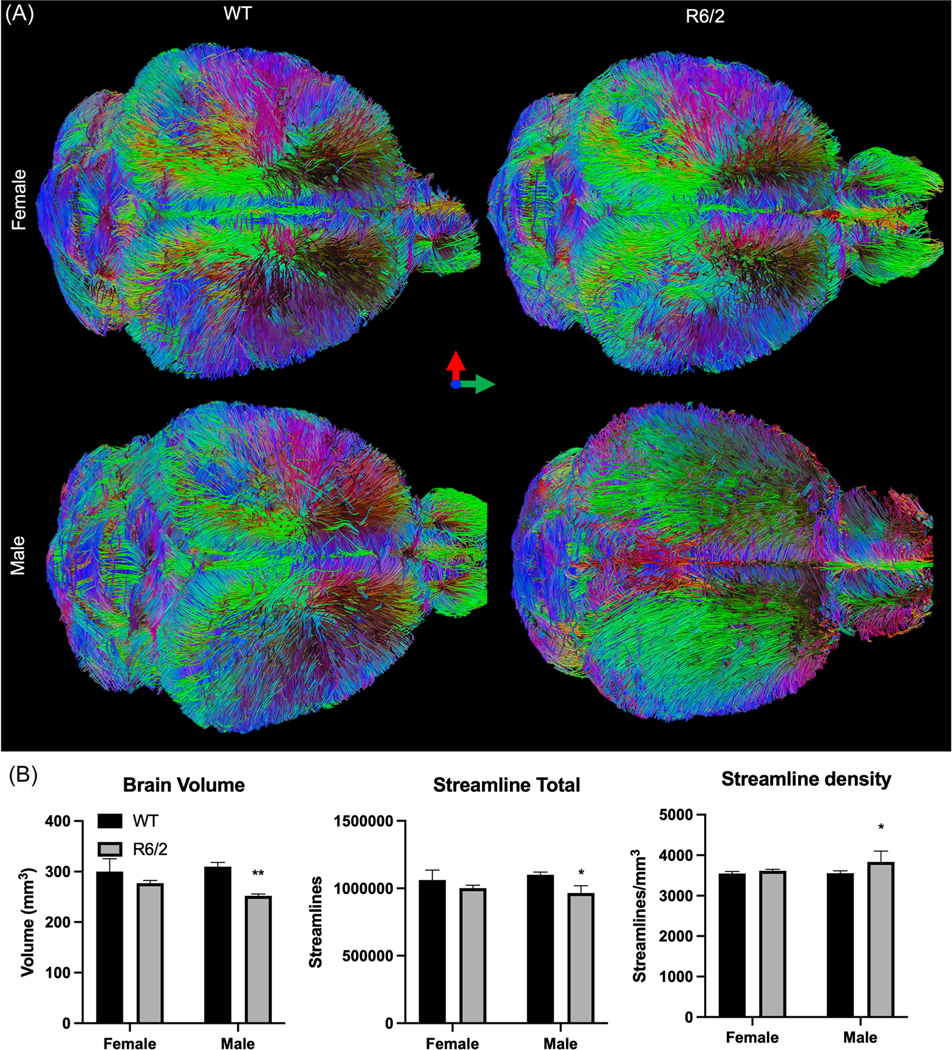
Whole brain volume and tractography. (A) Representative whole brain tractograms of WT and R6/2 male and female mice. (B) Whole brain volume was significantly reduced in male R6/2 mice, but not in female R6/2 mice. This difference was also evident in the total number of streamlines for whole brain. By considering brain volume and calculating streamline density, male R6/2 mice exhibited a higher streamline density, whereas female R6/2 mice had an equivalent streamline density compared to WT controls. *p ≤ 0.05, **p ≤ 0.01. WT, wild-type.

To assess the overall impact of HD, whole brain tractograms were generated to compare global volumetric changes and their impact on brain connectivity (Figure 3A). Only in male R6/2 did the disease process impact both brain volume and connectivity (Figure 3B). Total brain volume (*p* < 0.01) and number of streamlines (*p* < 0.05) were significantly reduced in male R6/2 mice but these did not reach statistical significance in female R6/2 mice. In contrast, when accounting for tissue atrophy, streamline density (i.e., streamlines per cubic millimeter of brain tissue) was actually significantly increased in male R6/2 mice (*p* < 0.05), potentially reflecting tissue atrophy without a commensurate loss of connectivity.

However, these global measures can mask or average-out tract-based changes in connectivity, especially if the affected regions are only a small proportion of the total brain volume. Quantitating streamlines for each ROI hence provides a more detailed and specific measure of connectivity changes that are impacted by neurodegeneration. Indeed, the total number of streamlines generated in striatum, GP, and STN were significantly reduced (*p* < 0.05) in both female and male R6/2 mice (Table 4, full statistical results can be found in Supporting Information: Table S4). Reductions in streamlines in female R6/2 mice (*p* < 0.05) were evident in the Ctx, MC, SMC, SN, thalamus, and HC, whereas the streamlines in the NAc and OB were only reduced in male R6/2 mice (*p* < 0.05). To account for volumetric changes in R6/2, streamline density was calculated as a “volume-independent” index of connectivity changes (Table 4). Streamline density was higher (*p* < 0.05) for male R6/2 mice in the Ctx, GP, and NAc, whereas female R6/2 mice exhibited a reduction in the striatum and HC (*p* < 0.05).

### 3.4 | Regional connectivity

**FIGURE 4:**
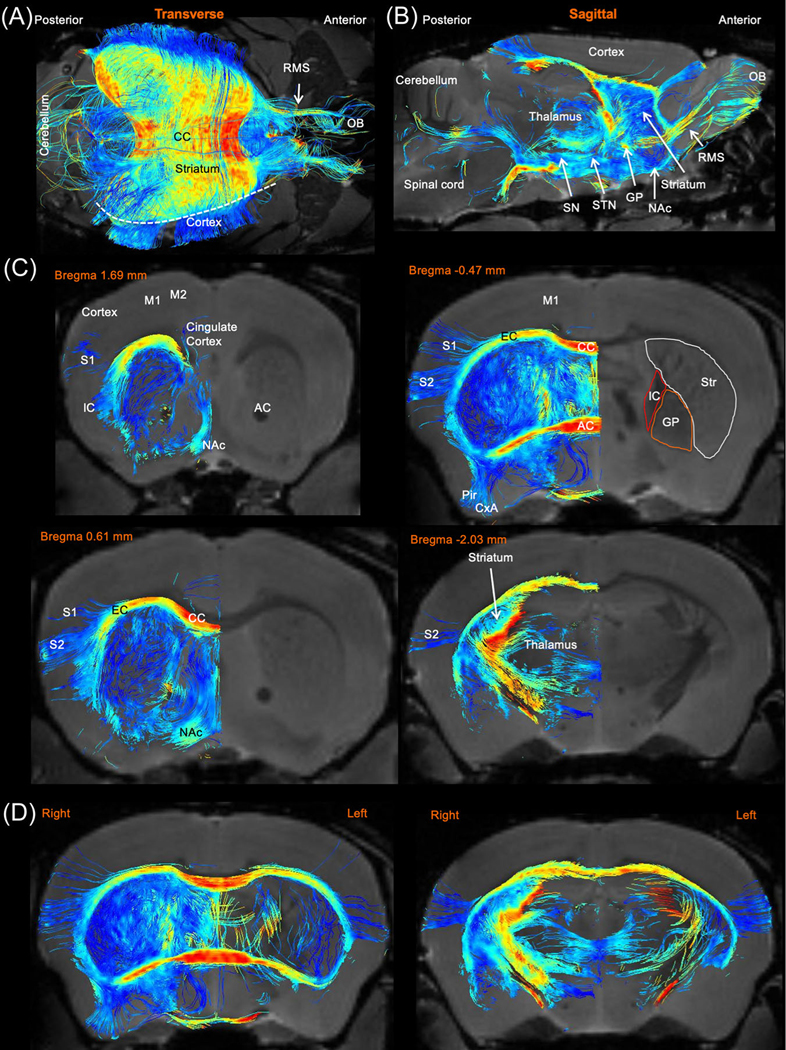
Striatal tractography. (A) Transverse connectivity of the R6/2 male striatum reveals extensive streamlines crossing the CC, projecting through the RMS into the OB, as well as the cerebellum. (B) A sagittal cut further details the subcortical connectivity of the striatum with the thalamus, GP, NAc, STN, and SN. (C). A coronal view in contrast provides a multislice view of how streamlines from the striatum connect with the SMC S1 and S2. (D) Transcallosal connectivity of the striatum highlights the complex interaction between both hemispheres. AC, anterior commissure; CC, corpus callosum; EC, external capsule; GP, globus pallidus; IC, internal capsule; NAc, nucleus accumbens; OB, olfactory bulb; RMS, rostral migratory stream; SMC, somatosensory cortex; SN, substantia nigra; STN, subthalamic nucleus.

The generation of streamlines from individual anatomical regions further afforded a connectivity analysis between two regions. For instance, connectivity tracing of the striatum revealed transcallosal connectivity, as well as projections into the OB, Ctx, and cerebellum (Figure 4A). A sagittal view reveals the subcortical connectivity of the striatum with the thalamus, as well as other structures forming the basal ganglia, such as the GP, NAc, STN, and SN (Figure 4B). Striatal connections with the SMC were also readily detected along the anterior–posterior axis (Figure 4C). There is also a robust interhemispheric connectivity through the CC and the anterior commissure (Figure 4D).

**FIGURE 5:**
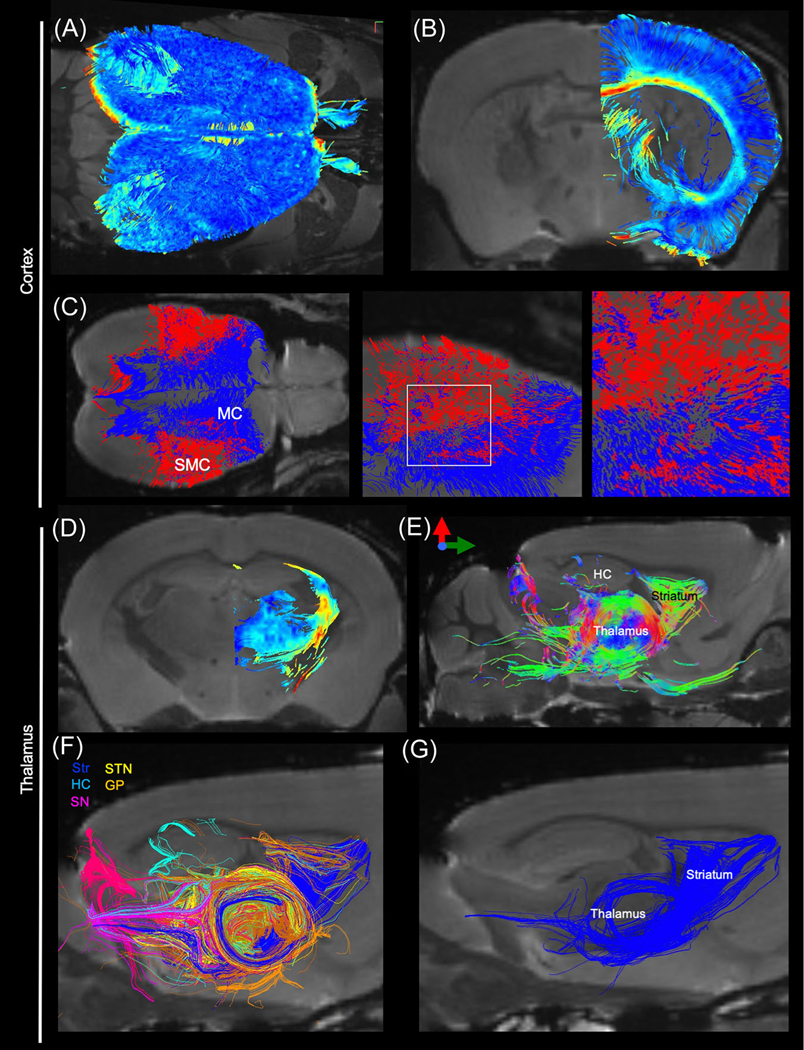
Tractography of the Ctx and thalamus. (A) Transverse view of streamlines seeded in the Ctx reveals widespread connectivity. (B) Streamlines project from the surface of the neocortex to the CC. (C) Parcelation of the streamlines seeded in the motor (blue) and somatosensory (red) cortex indicates mostly topographically separated territories, but with some notable areas of local overlap in both the motor and SMC. (D) Tractography of the thalamus. (E) Thalamic connectivity is highest with the striatum, but also projects to the HC. (F) Thalamic connectivity color-coded based on regions where connections are terminating. (G) Visualization of thalamo-striatal connectivity with seeds placed in the thalamus and selection of streamlines ending in the striatum. CC, corpus callosum; HC, hippocampus; SMC, somatosensensory cortex.

The Ctx generated vast amounts of streamlines across both hemispheres (Figure 5A) with directionality of the connections being perpendicular to the surface of the brain (Figure 5B). Segmentation into the MC and SMC highlights the separate topologies of these regions but also reveals their interconnectivity (Figure 5C). The thalamus also represents a key anatomical region involved in HD with projections to the HC (Figure 5D), but also widely connected to subcortical regions, including the striatum (Figure 5E). Color-coding of streamlines based on their regional connectivity emphasized the complexity of thalamic connections (Figure 5F). Parceling out of region-to-region connections affords a quantitative analysis of regional connectivity, such as thalamo-striatal pathways (Figure 5G).

**FIGURE 6:**
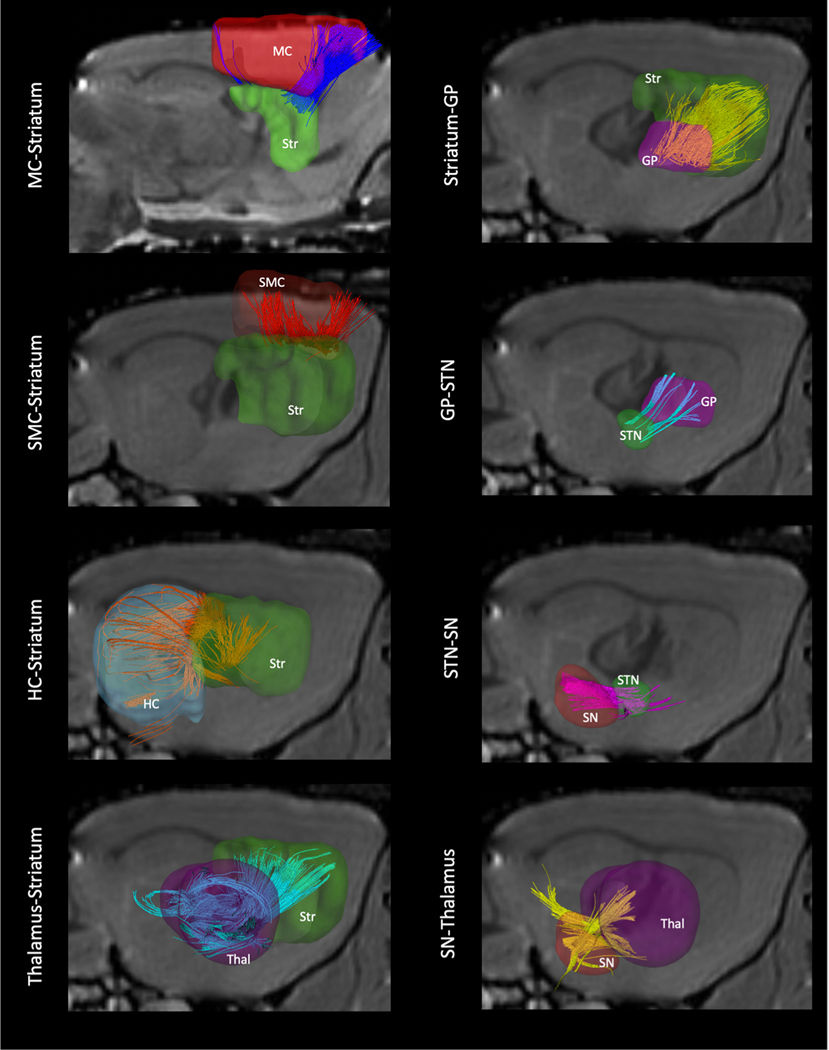
Regional tractography. Tracing of specific connections between two anatomical structures was achieved by seeding traces in one ROI and selecting those streamlines that ended in a target region. This allowed us to quantitatively assess the impact of Huntington’s disease on the connectivity of the basal ganglia. ROI, region-of-interest.

**FIGURE 7:**
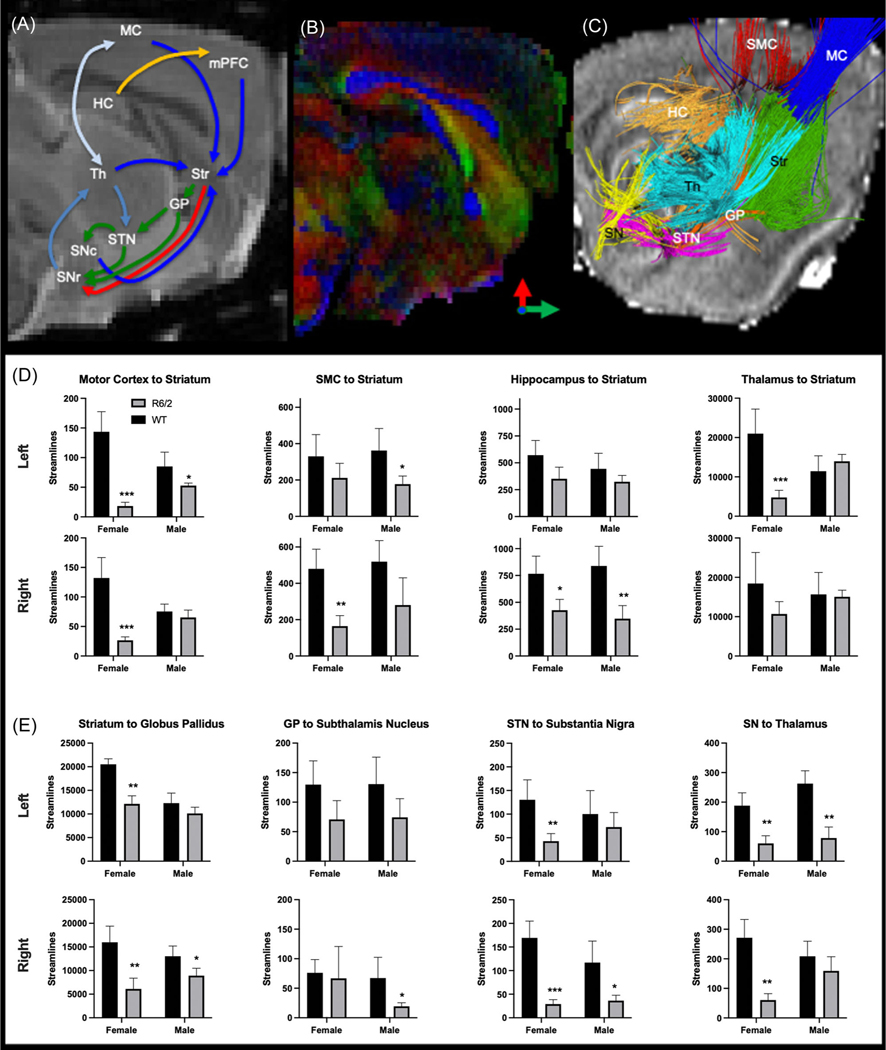
Tractographic dissection of the basal ganglia circuitry. (A) Schematic of the basal ganglia overlaid onto a sagittal *T*_2_-weighted female WT mouse. (B) DEC map reveals specific diffusion directionality in subcortical structures affording added contrast to distinguish small anatomical structures, such as the STN that are not easily segmented on *T*_2_-weighted images. (C) Tractogram of the basal ganglia. (D) Input connections to the striatum from the MC, and SMC are reduced in both male and female R6/2 mice. Only female R6/2 exhibited a significantly reduced connectivity of the thalamus with the striatum in the left hemisphere. The connections between the HC and the mPFC were significantly reduced in the left hemisphere in male R6/2 and the right hemisphere for female R6/2. (E) Output projections from the striatum to GP were also affected in female R6/2 mice, whereas in males this decrease was not significant. The magnitude of decrease in the right hemisphere of female R6/2 was also more pronounced than in the left hemisphere. The connection between the STN and SN was decreased in both male and female R6/2 mice in the right hemisphere, but only female R6/2 mice exhibited a significant decrease also in the left hemisphere. In contrast, in the left hemisphere the SN to thalamus connection was decreased in female and male R6/2 mice, but only female R6/2 mice had a significant decrease in the right hemisphere. *p ≤ 0.05, **p ≤ 0.01, ***p ≤ 0.001. DEC, diffusion-encoded color; GP, globus pallidus; HC, hippocampus; MC, motor cortex; mPFC, medial prefrontal cortex; SMC, somatosensory cortex; SN, substantia nigra; STN, subthalamic nucleus; WT, wild-type.

Maps of streamlines projecting from one region to another can hence be generated for each hemisphere (Figure 6). This region-to-region analysis affords an investigation of cortex-basal ganglia connectivity (Figure 7A). DEC images aided in the definition of subcortical structures (Figure 7B), such as the GP, to dissect the pathways connecting the basal ganglia and its connected structures (Figure 7C). Projections to the striatum were reduced for female (*p* < 0.001) and male R6/2 mice (*p* < 0.05) from the MC in the left hemisphere, whereas in the right hemisphere only female R6/2 mice were significantly affected (*p* < 0.001) (Figure 7D, full statistical results can be found in Supporting Information: Table S5). Connectivity between the SMC and striatum was variably affected in all R6/2 animals, but only the reduction in connectivity for male R6/2 mice in the left hemisphere (*p* < 0.05), and female R6/2 mice in the right hemisphere, reached statistical significance (*p* < 0.01). Hippocampal-medial prefrontal cortex projections were only significantly (*p* < 0.05) affected in the left hemisphere for male and the right hemisphere for female R6/2 mice. Thalamo-striatal connectivity only revealed a significant reduction (*p* < 0.001) for female R6/2 mice in the left hemisphere. Striato-pallidal connectivity, and connectivity between the STN and SN, as well as that between the SN and thalamus, were also impacted significantly in the right hemisphere for female (*p* < 0.01) and male R6/2 mice (*p* < 0.05), but only for female R6/2 mice (*p* < 0.01) in the left hemisphere (Figure 7E, full statistical results can be found in Supporting Information: Table S5). The pathway from the GP to STN was only significantly reduced in male R6/2 in the right hemisphere (*p* < 0.05). These results demonstrate how different pathways, in either hemisphere, are impacted by HD.

## 4 | DISCUSSION

As the brain is a highly interconnected organ, any loss of neurons will impact connectivity. We here demonstrate the utility of tractography in mapping connectivity changes in the basal ganglia and cortex, which form a network of interconnected regions that are progressively affected by HD. Biological sex impacted the magnitude and in some cases the direction of changes associated with the disease process. The magnitude of pathological changes between the left and right hemispheres was not equivalent for all measurements, indicating that disease progression may not be equivalent in each hemisphere.

### 4.1 | Diffusion MRI as a biomarker for HD

Brain atrophy in HD, as a consequence of neurodegeneration, has been extensively documented and can serve as a biomarker of disease progression in patients, as well as animal models.^24–26^ However, volumetric changes pertain to the overall structure and do not account for subtle structural or microstructural changes within that volume, which are expected to precede macroscopic volumetric changes. Voxel-based morphometry (VBM) or deformation-based morphometry (DBM) can localize subtle increases or decreases within an anatomical structure.^25,26,45,46^ However, group-based analyses and challenges in the standardization of image processing limit the diagnostic utility of VBM and DBM as a biomarker of HD in a clinical setting.^47^

Diffusion MRI can report on microstructural changes caused by neuronal loss, demyelination, and axonal injury, as well as extracellular space.^48^ Diffusion MRI hence reports on different biological substrates affected by HD and provides a unique tool to measure the impact of therapeutic interventions.^8^ As most therapeutics are first evaluated in transgenic animals modeling HD, such as R6/2 mice, we here characterized diffusion-based MRI changes, in addition to volume measurements, at the end-stage of the disease. It is expected that the magnitude of changes evident at this time point will provide the most robust experimental effects for a group comparison. The richness of diffusion measures (e.g., FA, MD) available through DTI position it as a unique assay with potentially multiple biomarkers that can report on different aspects of the disease condition (e.g., cell loss, axonal loss, myelin damage).

FA describes the degree of anisotropy of water movement within a voxel. FA here was not significantly different in R6/2 and WT control mice. This is consistent with a previous report that noted no difference in FA in the CC of *Hdh*Q150 mice, despite a significant brain atrophy.^32^ In contrast to FA, MD in the striatum was increased in female R6/2 mice and decreased in male R6/2 mice. MD is thought to be inversely related to cell density in tissues (i.e., high mean diffusion reflects lower cell density),^49^ potentially indicating that in female R6/2 mice cell density was higher than normal due to a compacting of tissue. In contrast, in male R6/2 mice, a decrease in MD is associated with a loss of cells, which is characteristic in neurodegenerative disease. We previously observed a neuron density increase in end-stage female and male R6/2,^24^ but MD is not specific to neurons and could hence indicate the loss of another cell type in male R6/2. Considering the same pattern of diffusion changes in RD and AD, which are mainly associated with changes in axonal density and myelination, diffusion changes here could, at least partially, be due to a loss of oligodendrocytes and axon degradation. A myelin breakdown has been observed in patients with HD, as evidenced by diffusion MRI,^50^ and has been demonstrated to occur before neuronal loss in transgenic mice.^33^

### 4.2 | Cortical and basal ganglia connectivity changes caused by HD

Connectivity analyses potentially can provide prodromal biomarkers that precede volumetric changes, as well as symptom onset.^51^ Establishing objectively measurable and robust prodromal biomarkers is essential to guide early interventions aimed at changing the disease onset.^52^ The dynamics of connectivity changes could hence provide a novel means to evaluate the impact of HD, as well as how interventions affect its progression.^13^ However, often tractography is focused solely on white matter connectivity (i.e., CC), neglecting connectivity changes within gray matter, such as changes in fiber tracts between the cortex and caudate-putamen/striatum, or within the basal ganglia. A limited number of studies demonstrated, nevertheless, that tractography can map changes in cortico-striatal connectivity to provide a detailed analysis of which regions are affected first by HD,^53^ whereas tractography analysis of the cortico-spinal tract suggested a dying back of axons during the presymptomatic phase of the condition.^54^ Widespread changes in the basal ganglia-cortical structural connectivity were reported using voxel-based analysis of probabilistic tractography in premanifest patients,^55^ further highlighting the potential for connectivity analysis to provide measurable changes that precede volumetric atrophy.

We here demonstrated that ex vivo mesoscale resolution (100 μm isotropic) diffusion MRI provides high-quality datasets for tractography. Although total streamlines were reduced for male R6/2 mice, this was not the case for female R6/2 mice. While accounting for the smaller brain volume of male R6/2 mice, streamline density was higher than in WT control mice. Whole brain measurements can average out specific point-to-point decreases and hence might not be a metric sensitive to changes associated with HD. Indeed, a more detailed analysis of connections between cortical regions and the striatum here revealed a decreased connectivity in both female and male R6/2 mice. This is consistent with reports of a cortico-striatal dysfunction in patients and animal models.^56–58^

A reduced connectivity was also evident within the basal ganglia. Dissection of individual connections within this network provides a unique means to eventually map the progressive nature of the disease and how these changes impact behavioral dysfunction.^58^ Although there were some subtle differences between male and female R6/2 mice, these differences were less pronounced than for volumetric changes between both sexes. In transgenic HD rats, for instance, 17β-estradiol levels correlated with reduced number of DARPP-32 positive neurons in males,^59^ suggesting that sex hormones play a major neuroprotective role that can impact tissue atrophy, as well as the severity of behavioral impairments. In contrast, female HD patients were reported to experience more severe behavioral impairments.^60^ This could potentially indicate key biological differences between both sexes in that male sufferers exhibit a volumetric change in response to a loss of connectivity, whereas in female carriers this same change in connectivity does not lead to the volumetric change, but could lead to a greater functional deficit.

It was also evident here that some changes were more pronounced in one hemisphere than the other, which is also consistent with observations in patients with HD.^44^ Animal studies are ideally suited to investigate hemisphere effects, as transgenic animals will follow a similar disease trajectory. Hemispheric effects in patients might be further related to handedness (i.e., dominant hemisphere effects), which could be included in analyses as a covariate.^61^ Although the small number of animals in each condition here afforded the inference of significant effects of HD on connectivity due to large effects and small variances between transgenic mice, future studies would benefit from a larger cohorts of animals to strengthen the statistical power of analyses and afford a more detailed investigation of subtle changes in connectivity (e.g., GP to STN), as one would expect in subjects with a smaller genetic load or in the prodromal phase of the condition. Beyond the connectivity between two regions, it would be useful to map connectivity to subregional territories and determine if particular tracts are especially vulnerable in the early stages of the disease.^53^

### 4.3 | Challenges for translating ex vivo mesoscale diffusion MRI in rodent transgenic models to clinical applications

A key aspect of resolving tracts within the basal ganglia is an isotropic mesoscale resolution that robustly defines each anatomical structure, while having a sufficient number of gradient diffusion-encoding directions to distinguish crossing, kissing, or fanning fibers. We previously demonstrated this principle to investigate connectivity in the human cerebellum^62^ and HC.^39,63,64^ This approach is applied here to the mouse brain to visualize connectivity between regions affected by HD. The 100 μm isotropic resolution required to visualize basal ganglia connectivity in the mouse is currently only achievable using ex vivo MRI due to the protracted time required to acquire sufficient signal and diffusion directions to permit this type of analysis.

Higher resolution images,^65^ as well as more complex diffusion acquisition schemes,^66,67^ can be acquired to provide an even more detailed analysis of connectivity in the mouse brain ex vivo. Ideally, diffusion MRI is acquired longitudinally in vivo to afford a comparison between different time points charting the progressive nature of HD.^32^ However, acquiring complex diffusion paradigms in vivo for tractography at a mesoscale resolution to dissect the basal ganglia in mice remains currently out of reach. Implementation of compressed sensing,^68^ higher magnetic field strengths,^28,69^ and further developments in multichannel radiofrequency (rf) coil design^70^ will be required to advance this approach to a standard assay required to measure the impact of therapeutic interventions.

Achieving a 100 μm isotropic resolution in vivo in patients for diffusion MR imaging is currently not possible. However, the fiber pathways of the basal ganglia in humans are much larger than those in mice and hence a spatial resolution of 500 μm will allow a similar dissection of individual pathways. Clinical 7 T MRI set-ups with multichannel coils dramatically improve whole human brain signal acquisition, with spatial resolutions of 100 μm achievable ex vivo^71^ and 200 μm in vivo.^72^ It can therefore be expected that with some additional technical advances in rf coil design and implementation of compressed sensing, diffusion MRI will be able to further resolve structural, as well as functional, basal ganglia-cortex connectivity in vivo in patients with Huntington’s.^14,15,55^

A major advantage of in vivo human imaging is the longitudinal nature of large cohorts of patients.^73^ This will allow within- and across-group comparisons using differential tractography^74^ that potentially can map how specific connections are gradually lost due to disease progression^53^ and how these can be spared using therapeutic interventions. MR imaging is increasingly emerging as a key diagnostic tool in HD. Identifying prodromal biomarkers is essential to provide an evidence-based guidance to evaluate disease-modifying neuroprotective strategies. Classical histopathological studies will not afford this guidance. Histopathological studies of post-mortem tissue, combined with ex vivo MRI, are important to establish and validate the neurobiological underpinnings of MRI- based biomarkers.^24,38,75^ However, tractographic systems-wide changes revealed by DTI are challenging to document using histological techniques due to the sectioning process.^39,76^

## 5 | CONCLUSIONS

In this proof-of-principle study, ex vivo diffusion MRI here allowed us to reveal microstructural changes that underpin volumetric changes in HD. FA was mostly unchanged, but MD, RD, and AD were affected. Important volumetric differences in male and female R6/2 mice, modeling end-stage Huntington’s, were evident, as were some differences in the left and right hemispheres. However, the neurobiological underpinnings of these structural changes remain unclear. Tissue atrophy reflects a change in its constitutive parts, which include cells, axons, and extracellular matrix. We have previously demonstrated that in R6/2 mice, no neuronal loss occurred, but neuron density increased.^24^ Tractography further allowed us here to dissect, for the first-time, connectivity changes in between the cortex and striatum, as well as within the basal ganglia. Widespread changes in connectivity were evident in R6/2 mice compared to WT controls. A commensurate decrease in streamlines with tissue atrophy, as indicated by streamline density, here potentially suggest that a loss of axonal connectivity is being related to tissue atrophy. These results further support clinical studies and suggest that diffusion MRI and tractography can provide more detailed complimentary measures that could serve as biomarkers to define the clinical horizon in addition to volumetric measurements. Quantitative in vivo biomarkers will be essential to measure the impact of neurotherapeutics that impact the progressive nature of HD. Ultimately, it is expected that a multitude of imaging biomarkers will be required to fully describe the disease course of HD.^77,78^

## Supplementary Material

Supplementary Figure 2

Supplementary Figure 1

Supplementary Figure 3

Supplementary Figure 4

Supplementary Figure 5

Supplementary Tables

## Figures and Tables

**TABLE 1:** Regional atrophy in male/female WT control and R6/2 Huntington’s disease mice

Regions	Volume in female (mm^3^)		Volume in male (mm^3^)	
	
	WT	R6/2	WT	R6/2
CCtx	55.77 ± 2.38	48.07 ± 1.31*	55.48 ± 2.30	49.08 ± 1.66
MC	6.66 ± 0.15	5.78 ± 0.09*	7.45 ± 0.65	6.79 ± 0.59
SMC	4.95 ± 0.14	4.10 ± 0.04*	5.94 ± 0.50	4.18 ± 0.19*
Str	7.95 ± 0.27	4.89 ± 0.40*	7.39 ± 0.25	5.78 ± 0.32*
GP	0.87 ± 0.04	0.72 ± 0.03*	0.70 ± 0.02	0.74 ± 0.02
SN	0.77 ± 0.01	0.54 ± 0.02*	0.68 ± 0.04	0.59 ± 0.01
NAc	0.6 ± 0.30	0.43 ± 0.03	0.81 ± 0.06	0.89 ± 0.09
Thal	3.29 ± 0.11	2.36 ± 0.10*	2.77 ± 0.17	2.62 ± 0.28
STN	0.14 ± 0.01	0.09 ± 0.10*	0.15 ± 0.01	0.12 ± 0.01*
HC	10.77 ± 0.85	7.92 ± 0.61*	9.52 ± 0.65	8.44 ± 0.92
OB	19.12 ± 2.37	19.69 ± 0.48	22.56 ± 0.66	19.52 ± 0.97*
CC	1.13 ± 0.06	1.00 ± 0.05	1.07 ± 0.06	1.02 ± 0.06

*Note*: Data were presented by mean ± standard deviation. Volumetric analysis of different brain regions revealed a reduced volume in both female and male R6/2 mice compared to controls in the CCtx, SMC, Str, SN, and STN. In contrast, a decreased volume only in female R6/2 mice was evident in the GP, MC, NAc, Thal, and HC. The OB volume of male R6/2 mice was reduced compared to controls. The CC did not differ between controls and diseased mice.

Abbreviations: CC, corpus callusum; CCtx, cerebral cortex; GP, globus pallidus; HC, hippocampus; MC, motor cortex; NAc, nucleus accumbens; OB, olfactory bulb; SMC, somatosensory cortex; SN, substantia nigra; STN, subthalamic nucleus; Str, striatum; Thal, thalamus; WT, wild-type.

* *p* ≤ 0.05.

**TABLE 2:** Regional measurements of scalar indices of diffusion (mm^2^/s)

Measurements	MD				AD				RD			
		
	Female		Male		Female		Male		Female		Male	
					
	WT	R6/2	WT	R6/2	WT	R6/2	WT	R6/2	WT	R6/2	WT	R6/2
CCtx	0.283 ± 0.008	0.290 ± 0.004	0.299 ± 0.011	0.268 ± 0.005*	0.351 ± 0.010	0.361 ± 0.007	0.368 ± 0.018	0.328 ± 0.008*	0.249 ± 0.007	0.254 ± 0.003	0.264 ± 0.008	0.238 ± 0.003*
MC	0.285 ± 0.008	0.295 ± 0.007	0.300 ± 0.009	0.273 ± 0.012*	0.352 ± 0.011	0.369 ± 0.012	0.375 ± 0.018	0.336 ± 0.016*	0.251 ± 0.007	0.258 ± 0.006	0.269 ± 0.007	0.241 ± 0.011*
SMC	0.274 ± 0.009	0.282 ± 0.006	0.293 ± 0.008	0.259 ± 0.007*	0.333 ± 0.012	0.344 ± 0.009	0.356 ± 0.016	0.310 ± 0.010*	0.244 ± 0.008	0.252 ± 0.005	0.265 ± 0.009	0.233 ± 0.006*
Str	0.266 ± 0.008	0.289 ± 0.002*	0.271 ± 0.011	0.246 ± 0.011*	0.323 ± 0.011	0.346 ± 0.002	0.324 ± 0.014	0.293 ± 0.012	0.238 ± 0.007	0.261 ± 0.003*	0.245 ± 0.010	0.223 ± 0.010*
GP	0.264 ± 0.015	0.277 ± 0.003	0.257 ± 0.010	0.248 ± 0.004	0.331 ± 0.015	0.353 ± 0.003	0.336 ± 0.014	0.321 ± 0.011	0.214 ± 0.009	0.237 ± 0.003*	0.218 ± 0.009	0.283 ± 0.008
SN	0.265 ± 0.010	0.282 ± 0.004	0.272 ± 0.013	0.253 ± 0.006*	0.355 ± 0.016	0.383 ± 0.006	0.364 ± 0.022	0.333 ± 0.010	0.224 ± 0.007	0.231 ± 0.003	0.226 ± 0.009	0.213 ± 0.004
NAc	0.264 ± 0.018	0.294 ± 0.002*	0.277 ± 0.013	0.267 ± 0.002	0.331 ± 0.015	0.368 ± 0.003*	0.339 ± 0.019	0.327 ± 0.003	0.247 ± 0.003	0.258 ± 0.002	0.246 ± 0.010	0.237 ± 0.003
Thai	0.261 ± 0.007	0.279 ± 0.002	0.353 ± 0.011	0.325 ± 0.006*	0.335 ± 0.008	0.363 ± 0.002*	0.353 ± 0.011	0.325 ± 0.006	0.224 ± 0.006	0.238 ± 0.002	0.242 ± 0.003	0.213 ± 0.004*
STN	0.270 ± 0.008	0.294 ± 0.002*	0.264 ± 0.008	0.270 ± 0.005	0.375 ± 0.010	0.406 ± 0.004*	0.373 ± 0.013	0.365 ± 0.010	0.217 ± 0.007	0.238 ± 0.003*	0.210 ± 0.007	0.235 ± 0.012*
HC	0.307 ± 0.007	0.331 ± 0.004	0.332 ± 0.008	0.305 ± 0.005*	0.393 ± 0.011	0.432 ± 0.005*	0.411 ± 0.015	0.385 ± 0.012	0.264 ± 0.006	0.281 ± 0.004*	0.293 ± 0.004	0.264 ± 0.003*
OB	0.301 ± 0.005	0.330 ± 0.016*	0.300 ± 0.017	0.312 ± 0.031	0.411 ± 0.006	0.453 ± 0.022	0.411 ± 0.031	0.412 ± 0.041	0.246 ± 0.005	0.268 ± 0.013*	0.245 ± 0.010	0.261 ± 0.026
CC	0.217 ± 0.004	0.219 ± 0.005	0.209 ± 0.009	0.193 ± 0.007*	0.388 ± 0.008	0.392 ± 0.015	0.371 ± 0.019	0.330 ± 0.013*	0.130 ± 0.003	0.133 ± 0.002	0.128 ± 0.004	0.124 ± 0.004

*Note*: Data were presented by mean ± standard deviation. MD was reduced in male R6/2 mice for the entire CCtx, MC, SMC, Str, and HC, whereas female R6/2 mice revealed an MD increase for the striatum. RD was significantly decreased in male R6/2 mice in the Ctx, MC, SMC, striatum, Thal, and HC. In contrast, in female R6/2, RD was increased in the striatum, GP, HC, and OB. RD was increased in both female and male R6/2 mice in the STN. AD was decreased in male R6/2 mice for the Ctx, motor and SMC, striatum, and CC. In female R6/2 mice, AD was increased in the NAc, thalamus, STN, and HC.

Abbreviations: AD, axial diffusivity; CC, corpus callosum; CCtx, cerebral cortex; GP, globus pallidus; HC, hippocampus; MC, motor cortex; MD, mean diffusivity; NAc, nucleus accumbens; OB, olfactory bulb; RD, radial diffusivity; SMC, somatosensory cortex; STN, subthalamic nucleus; Str, striatum; Thal, thalamus.

* *p* ≤ 0.05.

**TABLE 3:** Regional FA measurements

Measurements	Female		Male	
	
	WT	R6/2	WT	R6/2
CCtx	0.224 ± 0.007	0.229 ± 0.007	0.220 ± 0.013	0.214 ± 0.007
MC	0.232 ± 0.012	0.231 ± 0.014	0.232 ± 0.019	0.214 ± 0.004
SMC	0.204 ± 0.004	0.203 ± 0.010	0.199 ± 0.010	0.183 ± 0.004
Str	0.210 ± 0.004	0.198 ± 0.005	0.197 ± 0.005	0.194 ± 0.005
GP	0.284 ± 0.010	0.266 ± 0.006	0.288 ± 0.009	0.277 ± 0.030
SN	0.314 ± 0.009	0.335 ± 0.011	0.315 ± 0.014	0.296 ± 0.012
NAc	0.224 ± 0.005	0.239 ± 0.004	0.215 ± 0.011	0.218 ± 0.008
Thal	0.278 ± 0.010	0.296 ± 0.006	0.262 ± 0.015	0.294 ± 0.006
STN	0.388 ± 0.007	0.378 ± 0.010	0.391 ± 0.018	0.351 ± 0.012
HC	0.261 ± 0.009	0.278 ± 0.004	0.225 ± 0.015	0.248 ± 0.021
OB	0.324 ± 0.006	0.335 ± 0.013	0.317 ± 0.020	0.293 ± 0.007
CC	0.616 ± 0.014	0.617 ± 0.010	0.604± 0.015	0.576 ± 0.014

*Note*: Data were presented by mean ± standard deviation. No significant differences in FA were observed between controls and R6/2 mice for either biological sex.

Abbreviations: CC, corpus callosum; CCtx, cerebral cortex; FA, fractional anisotropy; GP, globus pallidus; HC, hippocampus; MC, motor cortex; NAc, nucleus accumbens; OB, olfactory bulb; SMC, somatosensory cortex; SN, substantia nigra; STN, subthalamic nucleus; Str, striatum; Thal, thalamus.

**TABLE 4:** Quantification of streamlines from individual regions

Regions	Streamlines’ total				Streamline density (/mm^3^)			
	
	Female		Male		Female		Male	
			
	WT	R6/2	WT	R6/2	WT	R6/2	WT	R6/2
CCtx	53,767.0 ± 2391.625	46,097 ± 1652.008*	477,744.0 ± 33745.908	50,524.667 ± 3944.609	965.274 ± 28.803	957.907 ± 12.434	863.691 ± 65.205	1066.446 ± 101.024*
MC	1160.750 ± 7.750	954.750 ± 32.126*	1631.500 ± 224.244	1316.0 ± 127.876	174.400 ± 2.656	165.222 ± 6.431	215.647 ± 13.558	193.824 ± 11.073
SMC	763.500 ± 26.285	622.500 ± 52.110*	1039.250 ± 136.233*	684.0 ± 75.825	154.715 ± 7.527	151.553 ± 11.387	172.422 ± 10.599	162.273 ± 12.644
Str	1998.250 ± 86.904	972.500 ± 160.619^†^	1889.250 ± 162.790	1409.750 ± 177.996*	251.334 ± 8.376	194.563 ± 16.270*	253.898 ± 14.240	242.089 ± 21.684
GP	63.250 ± 9.543	41.0 ± 3.559^†^	46.250 ± 3.544	33.500 ± 3.014*	71.549 ± 8.775	56.496 ± 3.533	47.610 ± 3.318	62.954 ± 6.427*
SN	24.250 ± 1.887	14.667 ± 2.603*	9.0 ± 2.739	19.250 ± 2.529	31.379 ± 2.985	27.129 ± 4.839	27.829 ± 3.609	32.136 ± 4.168
NAc	21.750 ± 1.250	15.0 ± 3.028	56.500 ± 14.925	37.250 ± 4.973*	36.314 ± 3.113	34.415 ± 5.265	45.627 ± 2.687	60.235 ± 10.105*
Thal	360.250 ± 26.078	246.500 ± 21.926*	284.0 ± 26.724	335.0 ± 64.855	109.286 ± 7.203	103.844 ± 5.226	101.671 ± 4.548	124.459 ± 16.311
STN	15.250 ± 2.594	6.667 ± 2.603*	11.250 ± 1.601	6.500 ± 2.327*	105.678 ± 16.150	66.536 ± 23.490	73.676 ± 11.067	54.857 ± 20.879
HC	3220.250 ± 369.015	2133.500 ± 174.697^†^	2572.250 ± 293.549	2494.333 ± 182.512	296.546 ± 11.152	269.249 ± 4.449*	267.712 ± 17.396	299.122 ± 21.155
OB	5442.0 ± 1097.349	5539.750 ± 113.500	6725.750 ± 290.435	5413.0 ± 325.577*	274.434 ± 35.696	281.496 ± 4.398	297.832 ± 6.658	324.350 ± 39.100
CC	105.750 ± 59.205	145.750 ± 28.173	132.0 ± 33.259	107.667 ± 40.168	86.945 ± 45.033	145.519 ± 28.936	125.788 ± 35.222	164.309 ± 61.046

*Note*: Data were presented by mean ± standard deviation The total number of streamlines emanating from the MC, SMC, Str, GP, and STN were reduced in both female and male R6/2 mice, whereas the whole CCtx, MC, SN, Thal, and HC only produced fewer streamlines in female R6/2 mice. Male R6/2 mice only exhibited decreased total streamlines in the OB and NAc. Streamline density accounts for volumetric difference between diseased and control subjects. A decrease in streamline density for females was evident in the striatum and HC, whereas an increase in streamline density in male R6/2 mice was revealed in the Ctx, globus pallidus and nucleus accumbens.

Abbreviations: CCtx, cerebral cortex; GP, globus pallidus; HC, hippocampus; MC, motor cortex; NAc, nucleus accumbens; OB, olfactory bulb; SMC, somatosensory cortex; SN, substantia nigra; STN, subthalamic nucleus; Str, striatum; Thal, thalamus; WT, wild-type.

* *p* ≤ 0.05.

† *p* ≤ 0.01.

## Data Availability

All MRI data will be made available through a data repository upon acceptance of the manuscript or through contacting the corresponding author.

## Highlights

- 100 mm isotropic resolution diffusion tensor imaging of the mouse brain for measurement of scalar indices and tractography.
- Whole brain and region-to-region connectivity measurements in wild type and R6/2 mice modelling Huntington’s disease.
