# Supplementary figures and images for: Ex vivo 100 μm isotropic diffusion MRI-based tractography of connectivity changes in the end-stage R6/2 mouse model of Huntington’s disease
Source: Neuroprotection. Author manuscript; Available in PMC 2023 Sep 22. (PMC10516267; doi:10.1002/nep3.14)

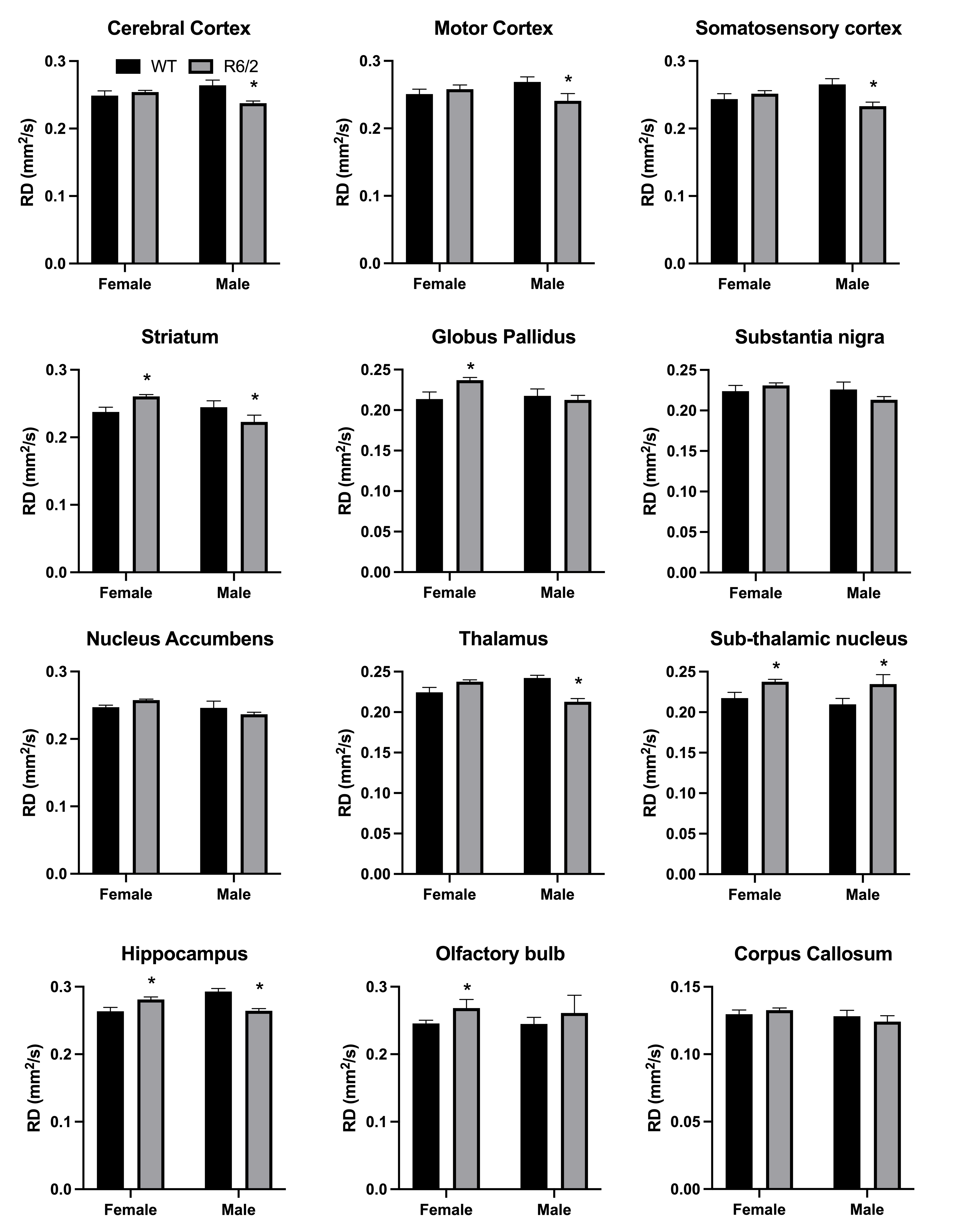

Supplement: Supplementary Figure 2 [file NIHMS1885818-supplement-Supplementary_Figure_2.tiff]

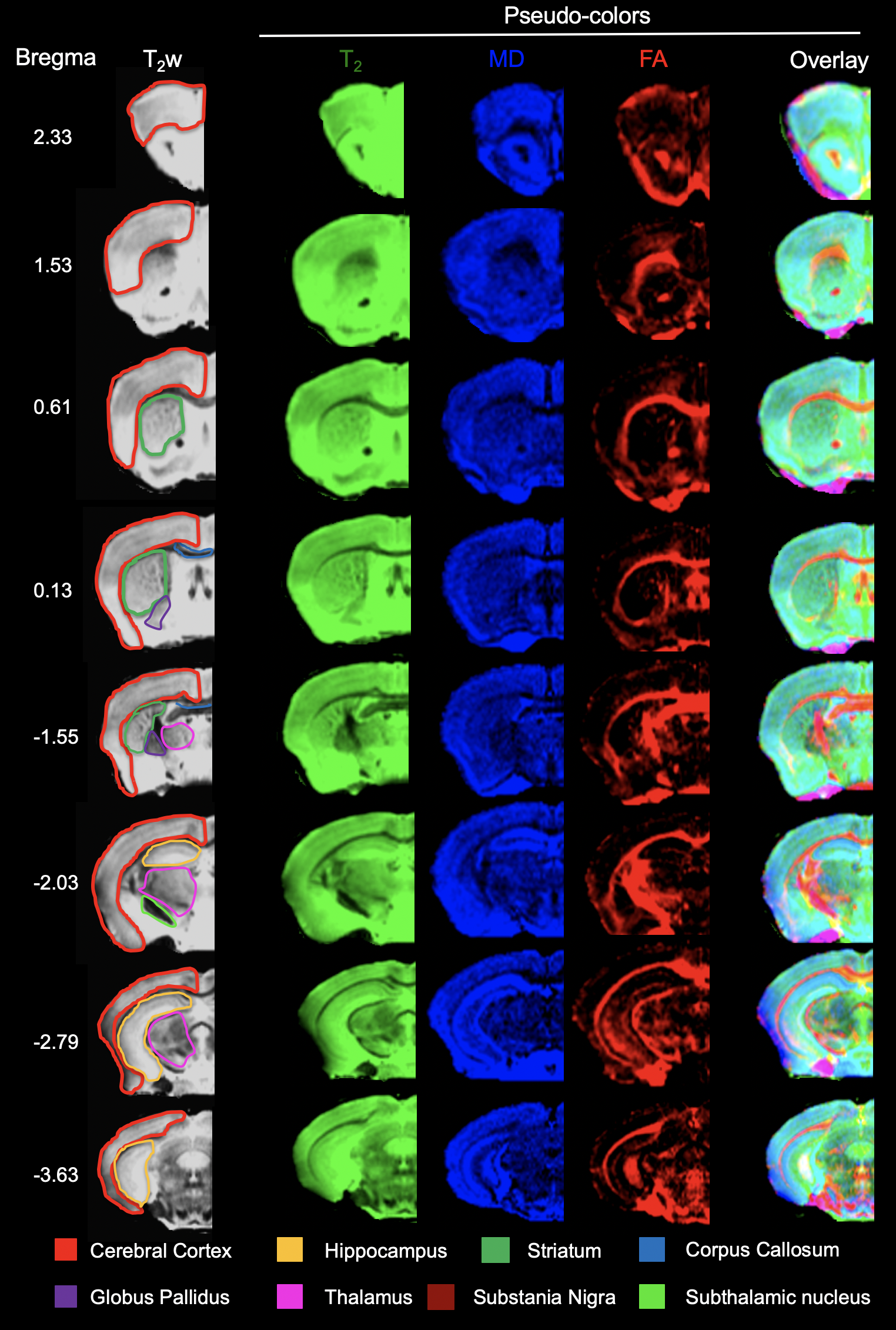

Supplement: Supplementary Figure 1 [file NIHMS1885818-supplement-Supplementary_Figure_1.tiff]

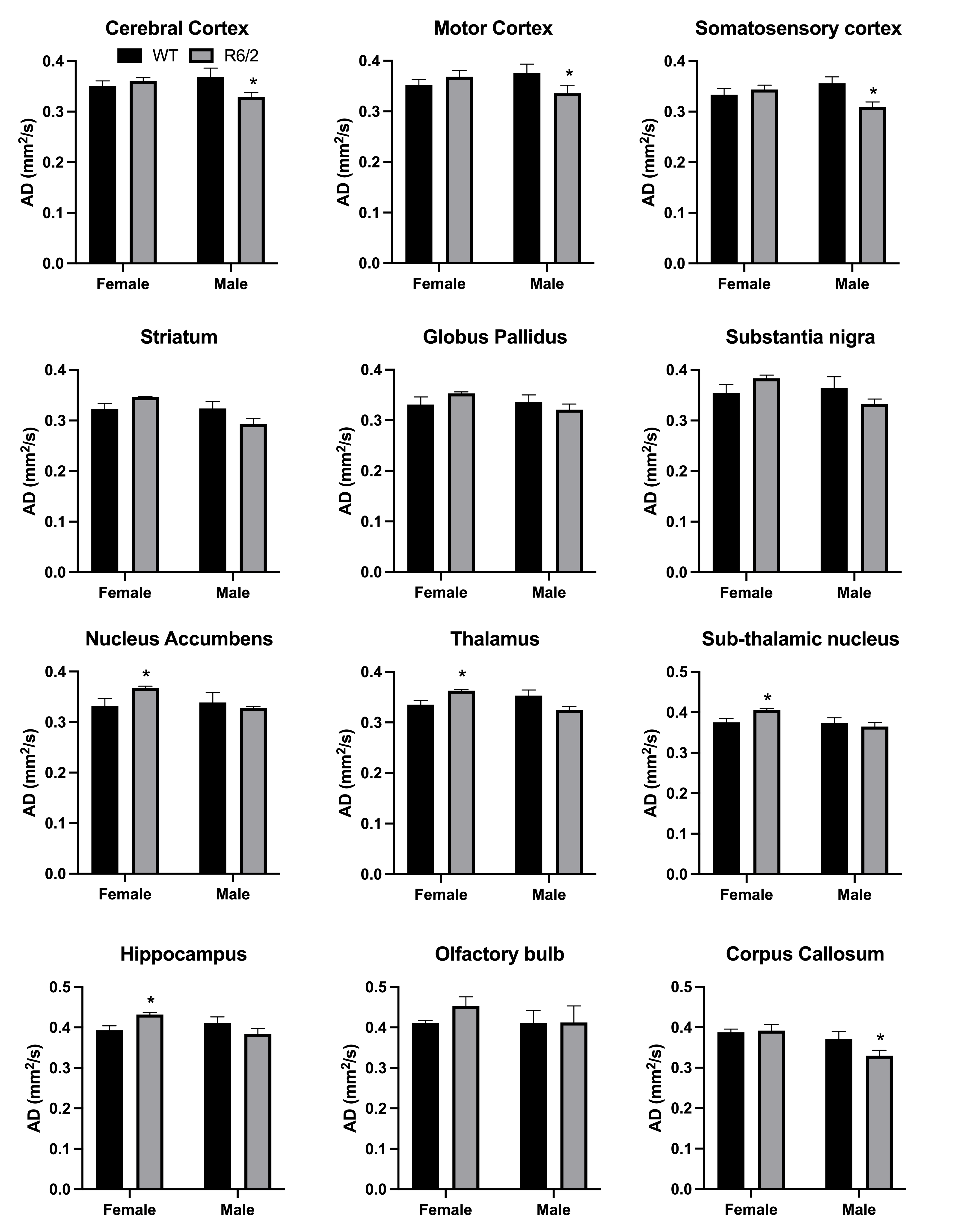

Supplement: Supplementary Figure 3 [file NIHMS1885818-supplement-Supplementary_Figure_3.tiff]

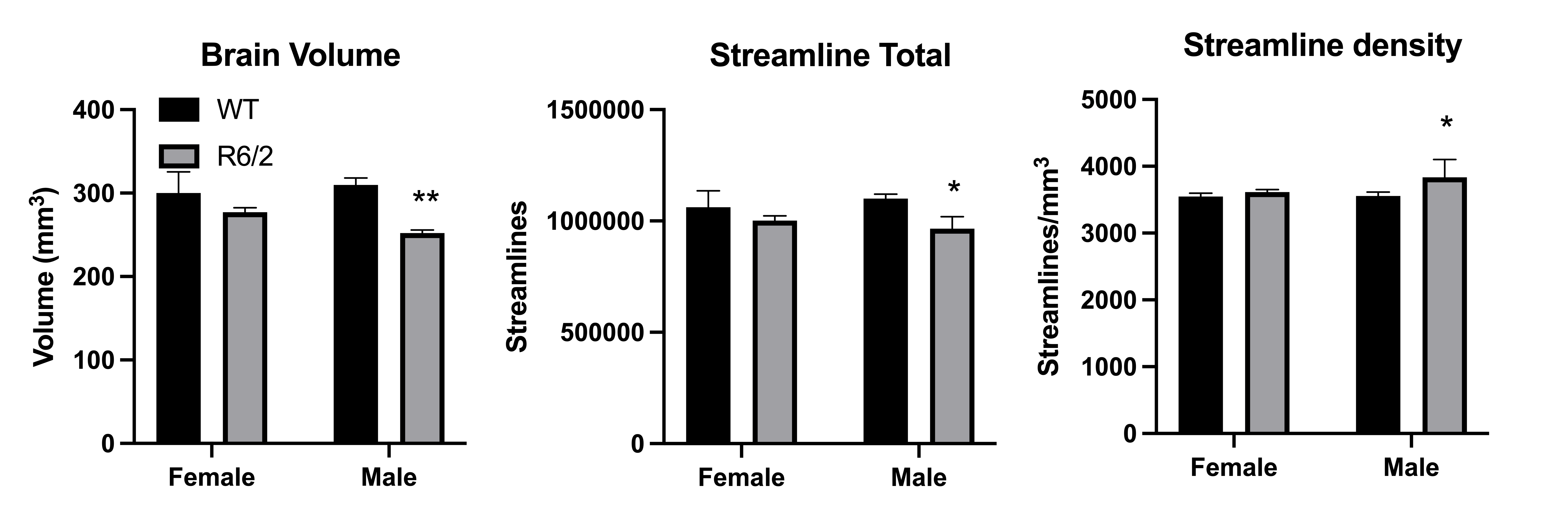

Supplement: Supplementary Figure 4 [file NIHMS1885818-supplement-Supplementary_Figure_4.tiff]

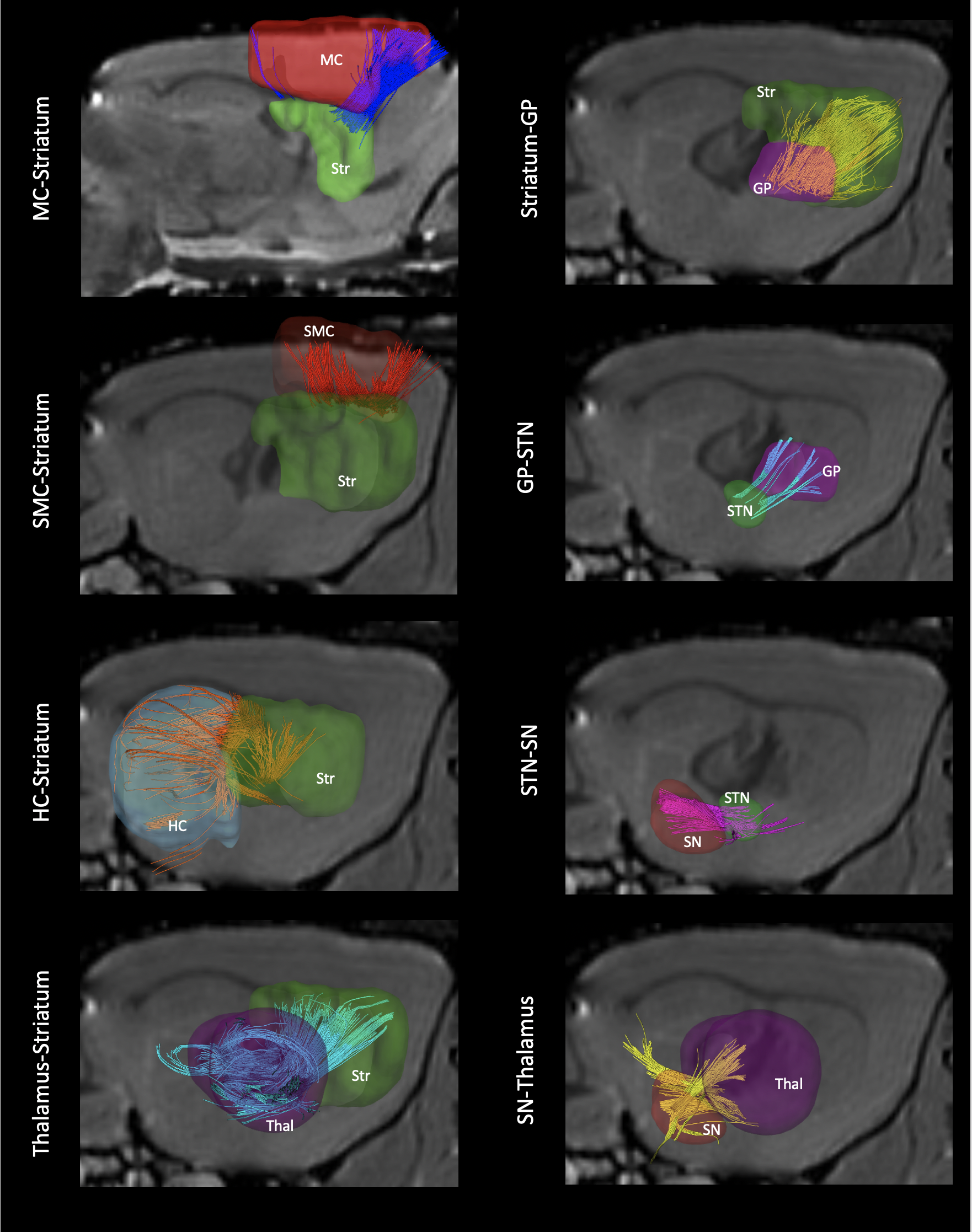

Supplement: Supplementary Figure 5 [file NIHMS1885818-supplement-Supplementary_Figure_5.tiff]
