# Supplementary material for: Ex vivo 100 μm isotropic diffusion MRI-based tractography of connectivity changes in the end-stage R6/2 mouse model of Huntington’s disease
Source: Neuroprotection. Author manuscript; Available in PMC 2023 Sep 22. (PMC10516267; doi:10.1002/nep3.14)
Supplement: Supplementary Tables [file NIHMS1885818-supplement-Supplementary_Tables.pdf]

Ex vivo 100 μm isotropic diffusion MRI-based tractography of connectivity changes in the end-stage R6/2 mouse model of Huntington's disease.

Ashwinee Manivannan[1], Lesley M. Foley[2], T. Kevin Hitchens[2,3], Ivan Rattray[4], Gillian P. Bates[4] & Michel Modo[5,6,7, CA]

University of Pittsburgh, [1]Department of Neuroscience, [2]Animal Imaging Center, [3]Department of Neurobiology, [5]Department of Radiology, [6]Department of Bioengineering, [7]Center for the Neural Basis of Cognition, Pittsburgh, PA15203, USA

[4]University College London, Queen Square Institute of Neurology, Department of Neurodegenerative Disease, Huntington's Disease Centre and UK Dementia Research Institute at UCL, London, WC1N 3BG, UK

Corresponding Author:

Dr Mike Modo
University of Pittsburgh
McGowan Institute for Regenerative Medicine
Department of Radiology
3025 East Carson Street
Pittsburgh, PA 15203
USA
Tel: +1 (412) 383 7200
E mail: mmm154@pitt.edu

Supplementary Tables

D.V.	**Volume (mm^3)**	
I.V.	Group	Sex
C.Ctx.	F(1;13)=13.750, p=0.0026	F(1;13)=0.034, p=0.8557
M.C.	F(1;13)=4.955, p=0.0420	F(1;13)=4.277, p=0.0591
S.M.C.	F(1;13)=19.148, p=0.0008	F(1;13)=3.261, p=0.0941
Str.	F(1;13)=40.683, p=0.0001	F(1;13)=0.197, p=0.6638
G.P.	F(1;13)=4.729, p=0.0481	F(1;13)=3.626, p=0.0793
S.N.	F(1;13)=24.415, p=0.0003	F(1;12)=0.621, p=0.4458
N.Ac.	F(1;13)=0.412, p=0.5318	F(1;13)=22.963, p=0.004
Thal.	F(1;13)=7.115, p=0.0194	F(1;13)=0.406, p=0.5347
S.T.N.	F(1;13)=22.649, p=0.0005	F(1;13)=5.446, p=0.0378
HC.	F(1;13)=6.947, p=0.0217	F(1;13)=0.312, p=0.5863
O.B.	F(1;13)=0.755, p=0.4005	F(1;13)=1.450, p=0.2500
C.C.	F(1;13)=2.396, p=0.1456	F(1;13)=0.141, p=0.7125

Supplementary Table 1. Statistical results for regional atrophy in male/female wild-type (WT) control and R6/2 Huntington's disease mice. Statistical results for the two independent variables (I.V.; Group= WT versus R6/2; Biological Sex = Male; Female) are reported for each outcome measure (i.e. dependent varible, D.V.).

Scalar Indices	MD (mm^2/s)		AD (mm^2/s)		RD (mm^2/s)	
**I.V.**	Group	Sex	Group	Sex	Group	Sex
**C.Ctx.**	F(1;13)=4.787, p=0.0475	F(1;13)=0.089, p=0.7699	F(1;13)=6.209, p=0.0270	F(1;13)=0.280, p=0.6055	F(1;13)=7.289, p=0.0182	F(1;13)=0.006, p=0.9349
**M.C.**	F(1;13)=4.697, p=0.0493	F(1;13)=0.107, p=0.7482	F(1;13)=5.495, p=0.0356	F(1;13)=0.078, p=0.7840	F(1;13)=6.248, p=0.0266	F(1;13)=0.001, p=0.9740
**S.M.C.**	F(1;13)=4.799, p=0.047301	F(1;13)=0.059, p=0.8111	F(1;13)=6.844, p=0.0213	F(1;13)=0.197, p=0.6639	F(1;13)=6.923, p=0.1888	F(1;13)=0.032, p=0.8606
**Str.**	F(1;13)=5.005, p=0.0434	F(1;13)=3.162, p=0.0988	F(1;13)=0.923, p=0.7660	F(1;13)=0.430, p=0.0583	F(1;13)=5.005, p=0.0207	F(1;13)=2.479, p=0.1394
**G.P.**	F(1;13)=0.0529, p=0.8216	F(1;13)=3.550, p=0.0821	F(1;13)=0.085, p=0.7747	F(1;13)=1.204, p=0.2925	F(1;13)=6.421, p=0.0249	F(1;13)=1.715, p=0.2130
**S.N.**	F(1;13)=6.0615, p=0.0285	F(1;13)=0.812, p=0.3852	F(1;13)=0.048, p=0.8294	F(1;13)=1.657, p=0.3225	F(1;13)=0.269, p=0.6131	F(1;13)=1.015, p=0.335
**N.Ac.**	F(1;13)=5.672, p=0.0332	F(1;13)=0.388, p=0.5440	F(1;13)=5.826, p=0.0312	F(1;13)=1.444, p=0.2509	F(1;13)=0.007, p=0.9330	F(1;13)=3.147, p=0.0874
**Thal.**	F(1;13)=5.311, p=0.03832	F(1;13)=52.874, p=0.0001	F(1;13)=5.002, p=0.9639	F(1;13)=0.868, p=0.3684	F(1;13)=6.265, p=0.0264	F(1;13)=0.255, p=0.6219
**S.T.N.**	F(1;13)=9.954, p=0.0075	F(1;13)=3.970, p=0.0696	F(1;13)=5.684, p=0.0434	F(1;13)=2.920, p=0.1132	F(1;13)=8.005, p=0.0152	F(1;13)=0.464, p=0.5086
**HC.**	F(1;13)=4.702, p=0.0492	F(1;13)=0.023, p=0.8808	F(1;13)=5.361, p=0.5590	F(1;13)=0.715, p=0.4142	F(1;13)=5.219, p=0.0397	F(1;13)=1.060, p=0.3235
**O.B.**	F(1;13)=5.158, p=0.0407	F(1;13)=0.251, p=0.6245	F(1;13)=0.622, p=0.0375	F(1;13)=0.546, p=0.4731	F(1;13)=6.700, p=0.0224	F(1;13)=0.070, p=0.7955
**C.C.**	F(1;13)=7.153, p=0.0191	F(1;13)=6.529, p=0.0240	F(1;13)=6.507, p=0.0241	F(1;13)=6.680, p=0.0227	F(1;13)=0.017, p=0.8963	F(1;13)=1.950, p=0.1860

Supplementary Table 2. Statistical results for regional measurements of scalar indices of diffusion. Statistical results for the two independent variables (I.V.; Group= WT versus R6/2; Biological Sex = Male; Female) are reported for each outcome measure (i.e. scalar indices).

D.V	FA	
**I.V.**	**Group**	**Sex**
**C.Ctx**	F(1;13)=0.002, p=0.9636	F(1;13)=1.411, p=0.2562
**M.C.**	F(1;13)=0.469, p-0.5055	F(1;13)=0.435, p=0.5209
**S.M.C.**	F(1;13)=1.309, p=0.2731	F(1;13)=2.859, p=0.1147
**Str.**	F(1;13)=3.104, p=0.1016	F(1;13)=3.262, p=0.0941
**G.P.**	F(1;13)=0.851, p=0.3729	F(1;13)=0.224, p=0.6436
**S.N.**	F(1;13)=0.001, p=0.9745	F(1;13)=1.891, p=0.1943
**N.Ac.**	F(1;13)=3.818, p=0.0726	F(1;13)=3.818, p=0.0726
**Thal.**	F(1;13)=6.126, p=0.0279	F(1;13)=0.829, p=0.3789
**S.T.N.**	F(1;13)=4.066, p=0.0667	F(1;13)=0.690, p=0.4222
**HC.**	F(1;13)=2.534, p=0.1374	F(1;13)=7.272, p=0.0194
**O.B.**	F(1;13)=0.256, p=0.6211	F(1;13)=3.256, p=0.0944
**C.C.**	F(1;13)=0.995, p=0.3366	F(1;13)=3.804, p=0.0730

Supplementary Table 3. **Statistical results for regional fractional anisotropy measurements.** Statistical results for the two independent variables (I.V.; Group= WT versus R6/2; Biological Sex = Male; Female) are reported for the dependent variable (i.e. FA).

D.V.	Streamlines Total		Stremline density (/mm^3)	
I.V.	Group	Sex	Group	Sex
C.Ctx	F(1;13)=4.835, p=0.465	F(1;13)=0.148, p=0.7068	F(1;13)=5.172, p=0.0405	F(1;13)=0.005, p=0.9409
M.C.	F(1;13)=5.287, p=0.0387	F(1;13)=10.913, p=0.0057	F(1;13)=2.828, p=0.1165	F(1;13)=14.365, p=0.0023
S.M.C.	F(1;13)=8.459, p=0.0122	F(1;13)=3.907, p=0.0697	F(1;13)=0.415, p=0.5307	F(1;13)=1.893, p=0.1922
Str.	F(1;13)=21.093, p=0.0005	F(1;13)=1.003, p=0.3349	F(1;13)=6.331, p=0.0257	F(1;13)=2.311, p=0.1524
G.P.	F(1;13)=9.986, p=0.0075	F(1;13)=4.893, p=0.0455	F(1;13)=0.0004, p=0.9840	F(1;13)=1.513, p=0.2405
S.N.	F(1;13)=7.415, p=0.0174	F(1;13)=0.066, p=0.8009	F(1;13)=0.008, p=0.9286	F(1;13)=0.014, p=0.9203
N.Ac.	F(1;13)=4.700, p=0.0493	F(1;13)=12.980, p=0.0032	F(1;13)=6.033, p=0.0288	F(1;13)=7.892, p=0.0148
Thal.	F(1;13)=5.113, p=0.0415	F(1;13)=0.019, p=0.8911	F(1;13)=0.754, p=0.4010	F(1;13)=0.423, p=0.5266
S.T.N.	F(1;13)=8.210, p=0.0142	F(1;13)=0.961, p=0.3462	F(1;13)=2.621, p=0.1314	F(1;13)=1.687, p=0.2184
HC.	F(1;13)=6.120, p=0.0279	F(1;13)=0.355, p=0.5622	F(1;13)=5.000, p=0.0435	F(1;13)=0.012, p=0.9146
O.B.	F(1;13)=4.754, p=0.0482	F(1;13)=0.986, p=0.3402	F(1;13)=0.421, p=0.5274	F(1;13)=1.641, p=0.2226
C.C.	F(1;13)=0.060, p=0.8100	F(1;13)=0.006, p=0.9359	F(1;13)=1.298, p=0.2751	F(1;13)=0.457, p=0.5107

Supplementary Table 4. **Statistical results comparing streamlines from individual regions**. Statistical results for the two independent variables (I.V.; Group= WT versus R6/2; Biological Sex = Male; Female) are reported for the dependent variable (i.e. streamlines total and density).

Hemisphere	Left		Right	
I.V.	Group	Sex	Group	Sex
M.C. to Str.	F(1;13)=10.639, p=0.0062	F(1;13)=0.246, p=0.6276	F(1;13)=5.634, p=0.0314	F(1;13)=0.345, p=0.5656
S.M.C. to Str.	F(1;13)=5.628, p=0.0337	F(1;13)=0.0001, p=0.9916	F(1;13)=6.457, p=0.0246	F(1;13)=0.508, p=0.4883
Thal. To Str.	F(1;13)=7.042, p=0.0198	F(1;13)=0.016, p=0.9001	F(1;13)=0.7187, p=0.4132	F(1;13)=0.054, p=0.8194
HC to mPFC	F(1;13)=9.885, p=0.0744	F(1;13)=0.253, p=0.6243	F(1;13)=7.233, p=0.0211	F(1;13)=5.186, p=0.0437
Str to G.P.	F(1;13)=7.367, p=0.0188	F(1;13)=6.896, p=0.0221	F(1;13)=8.174, p=0.0144	F(1;13)=0.003, p=0.9528
G.P. to S.T.N.	F(1;13)=2.511, p=0.1370	F(1;13)=0.003, p=0.9543	F(1;13)=5.672, p=0.0332	F(1;13)=0.547, p=0.4748
S.T.N. to S.N.	F(1;13)=6.470, p=0.0244	F(1;13)=0.0001, p=0.9947	F(1;13)=13.662, p=0.0027	F(1;13)=0.566, p=0.4651
S.N. to Thal.	F(1;13)=17.345, p=0.0011	F(1;13)=1.530, p=0.2380	F(1;13)=6.455, p=0.0246	F(1;13)=0.121, p=0.7327

Supplementary Table 5. **Statistical results for regional tractography**. Statistical results for the two independent variables (I.V.; Group= WT versus R6/2; Biological Sex = Male; Female) are reported for the dependent variable (i.e. streamlines) between two regions of interest.
